# Conservative versus liberal oxygen therapy for mechanically ventilated patients: a systematic review and meta-analysis of randomized controlled trials

**DOI:** 10.3389/fmed.2026.1697749

**Published:** 2026-04-24

**Authors:** Juan Liu, Jing Huang, Xiaohui Wang, Xiaomei Xu, Chao Huang, Daiqiang Liu, Junchen Zhu, Lvlin Chen, Xinwei Chen, Lin Chen, Dongjiao Dou, Xiangui Lv

**Affiliations:** 1 Department of Intensive Care Medicine, Affiliated Hospital of Chengdu University, Chengdu, Sichuan, China; 2 Chengdu Fourth People's Hospital, Chengdu, Sichuan, China; 3 Department of Nursing, Affiliated Hospital of Chengdu University, Chengdu, Sichuan, China; 4 Chengdu University, Chengdu, Sichuan, China

**Keywords:** conservative oxygen therapy, liberal oxygen therapy, mechanically ventilated patient, systematic review, meta-analysis

## Abstract

**Background:**

Currently, clinicians lack sufficient evidence to determine which oxygen therapy approach yields better outcomes in mechanically ventilated (MV) patients, whether conservative oxygen therapy (COT) or liberal oxygen therapy (LOT).

**Methods:**

This study systematically searched for randomized controlled trials (RCTs) in PubMed, Embase, Web of Science, and Cochrane Library from the inception of each database to 1 September 2025. The outcome measures in MV patients included overall mortality, intensive care unit (ICU) mortality, 90-day mortality, ICU length of stay (LOS), hospital LOS, and MV hours.

**Results:**

This meta-analysis included 11 RCTs published between 2014 and 2025, involving a total of 20,786 adult MV patients. There was no significant difference in overall mortality between the COT and LOT groups (RR 1.02, 95% CI 0.95–1.10; *Z* = 0.54, *p* = 0.59) nor in ICU mortality (RR 1.07, 95% CI 0.89–1.33; *Z* = 0.71, *p* = 0.48) and 90-day mortality (RR 1.04, 95% CI 0.96–1.12; *Z* = 0.92, *p* = 0.36). There was no significant difference in ICU LOS between COT and LOT (MD −0.02, 95% CI −0.05–0.01, *Z* = −1.42, *p* = 0.15), or in hospital LOS (MD 0.00, 95% CI −0.06–0.07, *Z* = 0.14, *p* = 0.89), and MV hours (MD −0.05, 95% CI −0.92–0.81, *Z* = −0.12, *p* = 0.91).

**Conclusion:**

In this meta-analysis of MV patients, COT was not associated with a reduction in overall mortality, ICU mortality, 90-day mortality, ICU LOS, hospital LOS, and MV hours when compared with LOT. The absence of overall benefit from COT in this broad population does not preclude the possibility that selected subgroups or different target ranges could prove advantageous.

**Systematic review registration:**

https://www.crd.york.ac.uk/PROSPERO/view/CRD420251137389.

## Background

Mechanical ventilation (MV) is a fundamental life-support intervention for critically ill patients in the intensive care unit (ICU). In-hospital mortality for critically ill adults receiving MV remains approximately 35% (1, 2). Oxygen therapy is essential for maintaining or improving oxygenation levels in MV patients (3). For every patient receiving MV, the fraction of inspired oxygen (FiO₂) must be titrated to maintain arterial oxygen saturation. LOT targeting higher oxygen saturation levels provides a safety margin against hypoxemia but may increase exposure to excessive hypoxemia and tissue hyperoxia (4). The excessive amount of oxygen in the blood and/or tissues is associated with adverse clinical outcomes of various diseases, including acute respiratory distress syndrome (ARDS), post-cardiac arrest syndrome, unexpected hypothermia, and stroke (5–8). Animal studies have shown that sustained arterial hypoxemia may lead to progressive lung injury, interstitial edema, and pulmonary inflammatory activation (3, 9, 10). COT targeting lower oxygen saturation levels may minimize the risk of hyperoxia but could increase the risk of hypoxemia and tissue hypoxia. Palmer et al. (7) showed that exposure to supraphysiological levels of oxygen could lead to increased mortality in critically ill patients. Clinicians lack robust evidence to determine whether the COT or LOT approach yields superior outcomes for MV patients.

Previous systematic reviews and meta-analyses (SRMA) have also reported on the effects of COT versus LOT and found that COT was not associated with overall mortality (11, 12). More recently, Dong et al. conducted a meta-analysis involving 7 RCTs, involving 1802 MV patients (11). Following this SRMA, several RCTs were published, and one of them was conducted in 97 ICUs in the UK, including 16,500 MV patients (13), exceeding the total sample size of the aforementioned SRMA (11, 12). Furthermore, the optimal peripheral oxygen saturation (SpO₂) target range for COT remains a subject of debate, with trials applying thresholds ranging from 88–92% to 90–98%. Whether the strictness of the oxygen target modifies the treatment effect has not been systematically examined. To address these gaps, we conducted an updated systematic review and meta-analysis incorporating all available RCTs. The main aim was to provide the most precise estimates of the effects of COT versus LOT on mortality and other clinical outcomes in MV adults. Additionally, this study performed predefined subgroup analyses according to the strictness of the COT to explore whether different SpO₂ thresholds influence outcomes.

## Methods

This meta-analysis was conducted in accordance with the Preferred Reporting Items for Systematic Reviews and Meta-Analyses (PRISMA) statement (14). The research protocol was registered in the International Prospective Register of Systematic Reviews (PROSPERO) with registration number CRD420251137389.

### Search strategies

This study conducted a systematic search of several databases such as PubMed, Embase, Web of Science, and Cochrane Library for RCTs published up to 1 September 2025. The search strategy included the following terms: “conservative oxygen therapy,” “COT,” “oxygenation target,” “liberal oxygen therapy,” “LOT,” “liberal oxygenation targets,” “conventional oxygen therapy,” “usual oxygen therapy,” “mechanical ventilation,” “randomized controlled trial, and “RCT.” The authors also manually searched the reference list of relevant review articles. The complete search strategy is provided in Supplementary material S1.

### Inclusion and exclusion criteria

Eligible clinical trials were identified based on the following criteria: (a) study design involved RCTs; (b) study population comprised adult patients receiving MV in the ICU; (c) intervention involved comparison of the effects of COT and LOT, where COT was defined as a target blood oxygen saturation level of 90–98% and LOT was defined as a higher oxygen saturation target; (d) primary outcomes included all-cause mortality, ICU mortality, and 90-day mortality; and secondary outcomes included ICU length of stay (ICU LOS), hospital LOS, and MV hours. The exclusion criteria were as follows: (a) reviews, case reports, and protocols and (b) research conducted on animals or patients under 18 years old.

### Literature screening and data extraction

Two investigators (XL and JL) independently performed literature screening and data extraction. In case of disagreement, a third researcher (CH) was consulted to reach a decision. The following variables were extracted: first author, year, country, sample size, demographic characteristics, oxygen therapy targets, and duration of intervention.

### Quality assessment

Two researchers (XL and JL) independently assessed the quality of included RCTs using the Cochrane Risk of Bias Assessment Tool (15). Disagreements were adjudicated by the third researcher (CH). The following criteria are indicators of the quality of trials: (a) sequence generation, (b) allocation concealment, (c) blinding of participants and personnel, (d) blinding of outcome assessment, (e) incomplete outcome data, (f) selective outcome reporting, and (g) other sources of bias.

### Statistical analysis

Statistical analysis was performed using Stata software (version 18.0). For dichotomous outcomes, the risk ratio (RR) and 95% confidence interval (CI) were calculated, while for continuous data, the mean difference (MD) and 95% CI were calculated. Heterogeneity across studies was assessed using the I^2^ statistic. This study evaluated the study-level heterogeneity using the I^2^ statistic, categorized as low (0–25%), moderate (26–50%), and high (>50%). In the presence of considered clinical heterogeneity and to ensure the reliability of the results, this study used a random-effects model for all outcomes. Subgroup analyses were conducted based on the strictness of COT targets (88–92% versus 90–98%). Publication bias was initially assessed by the visual inspection of funnel plot symmetry and further examined using Egger’s test for quantitative analysis. A two-sided *p*-value of <0.05 was considered statistically significant.

### Literature search

**Figure 1:**
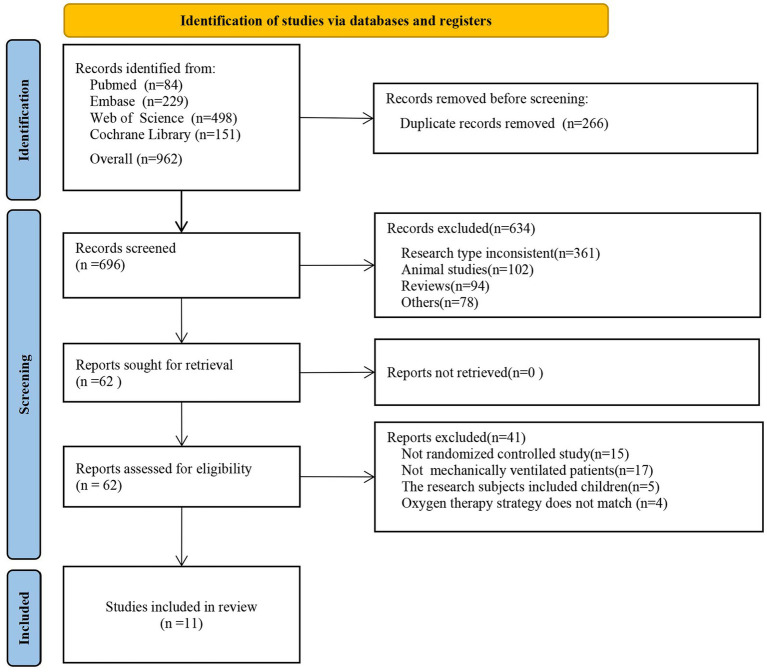
PRISMA study selection flowing chart.

The study identified 962 studies (84 from PubMed, 229 from Embase, 498 from Web of Science, and 151 from Cochrane Library) through a literature search, screened, and ultimately included 11 RCTs in the final analysis (13, 16–25). The comprehensive flowchart detailing the progress of the literature selection process is shown in Figure 1.

### Study characteristics

All 11 included RCTs were published between 2014 and 2025, involving a total of 20,786 adult MV patients. The characteristics of the included studies are shown in Table 1.

**Table 1:** Characteristics of included studies.

Author	Year	Country	Sample size, n	Multicenter	Patient type	Male, *n* (%)		Age, years		APACHE III score		SOFA score		Oxygen therapy targets		Duration of intervention
						COT	LOT	COT	LOT	COT	LOT	COT	LOT	COT	LOT	
Barrot et al. (17)	2020	France	205	YES	ARDS	65 (65.7)	64 (62.7)	63.0 ± 15.5	63.5 ± 14.5^a^	NA	NA	9.3 ± 3.68	8.9 ± 3.6	PO_2_ 55–70 mmHg or SpO_2_ 88–92%	PO_2_ 90–105 mmHg or SpO_2_ ≥ 96%	7 days
Ghazaly et al. (18)	2024	Egypt	106	NO	Sepsis	36 (67.9)	33 (62.3)	48.04 ± 12.25	52.60 ± 16.21	18.75 ± 9.28	16.42 ± 6.76	7.47 ± 2.92	7.49 ± 3.57	PO_2_ 60–75 mmHg or SpO_2_ 88–92%	PO_2_ 90–105 mmHg or SpO_2_ ≥ 96%	ICU discharge
Girardis et al. (19)	2016	Italy	434	NO	MV > 72 h	121 (56)	125 (42.7%)	63 (51–74)^a^	65 (52–76)^a^	NA	NA	NA	NA	PO_2_ 70–100 mmHg or SpO_2_ 94–98%	Allowing up to 150 mm Hg or SpO_2_ ≥ 97%	ICU discharge
Mackle et al. (20)	2020	Australia	965	YES	MV > 24 h	306 (63.2)	302 (62.8)	58.1 ± 16.2	57.5 ± 16.1	23.6 ± 9.3	23.3 ± 9.4	NA	NA	SpO_2_ 90–97%	no restrictions	28 days or ICU discharge
Martin et al. (13)	2025	The United Kingdom	16,500	YES	MV > 12 h	4,537 (61.8)	4,616 (61.8)	60 (48––71)^a^	60 (48–71)^a^	16 (12–21)^a^	16 (12–21)^a^	NA	NA	SpO_2_ 88–92%	no restrictions	90 days or ICU discharge
Panwar et al. (21)	2016	Australia	103	YES	MV > 24 h	32 (62)	33 (65)	62.4 ± 14.9	62.4 ± 17.4	NA	NA	7.9 ± 2.9	7. 4 ± 3.1	SpO_2_ 88–92%	≥96	Entire duration of mechanical ventilation
Suzuki et al. (22)	2014	Australia	105	NO	MV > 48 h	38 (74.5)	32 (59.2)	59 ± 17	56 ± 16	NA	NA	NA	NA	SpO_2_ 90–92%	Determined by clinical doctors	Free of mechanical ventilation for greater than 24 h, death, or up to 28 day
Young et al. (23)	2020	Australia	251	YES	Sepsis	75 (57.7)	59 (48.8)	58.3 ± 15	57.2 ± 14.3	22.7 ± 7.5	22.8 ± 8.2	NA	NA	SpO_2_ 90–97%	NA	14 days or ICU discharge
van der Wal et al. (24)	2023	Netherlands and Italy	664	YES	MV > 24 h	224 (66.9)	211 (64.1)^a^	67 (59–74)^a^	67(56–73)^a^	NA	NA	9 (7–11)^a^	9 (7–11)^a^	PO_2_ 55–80 mm Hg SpO_2_ 91–94%	PO_2_ 110–150 mm Hg SpO_2_ 96–100%	28 days or ICU discharge
Asfar et al. (16)	2017	France	434	YES	Septic shock	140 (65%)	137 (63%)	66.3 ± 14.6	67.8 ± 12.7	NA	NA	10.3 ± 2.9	10.2 ± 2.7	Spo_2_: 88–95%	Fio_2_ of 1.0 for 24 h after inclusion	24 h
Semler et al. (25)	2022	America	1,683	NO	MV	447 (55.3)	465 (53.2)	57 (44–67)	59 (45–68)	NA	NA	5 (4–8)	5 (3–8)	SpO_2_ 88–92%	SpO_2_ 96–100%	ICU discharge, Discontinue ventilator support or 2-month

APACHE, The Acute Physiologic and Chronic Health Evaluation; NA, not applicable; COT, conservative oxygen therapy; LOT, liberal oxygen therapy; SpO_2_, Pulse oxygen saturation; PaO_2_, partial pressure of arterial oxygen; TBI, traumatic brain injury.

a Median (IQR).

### Mortality

**Figure 2:**
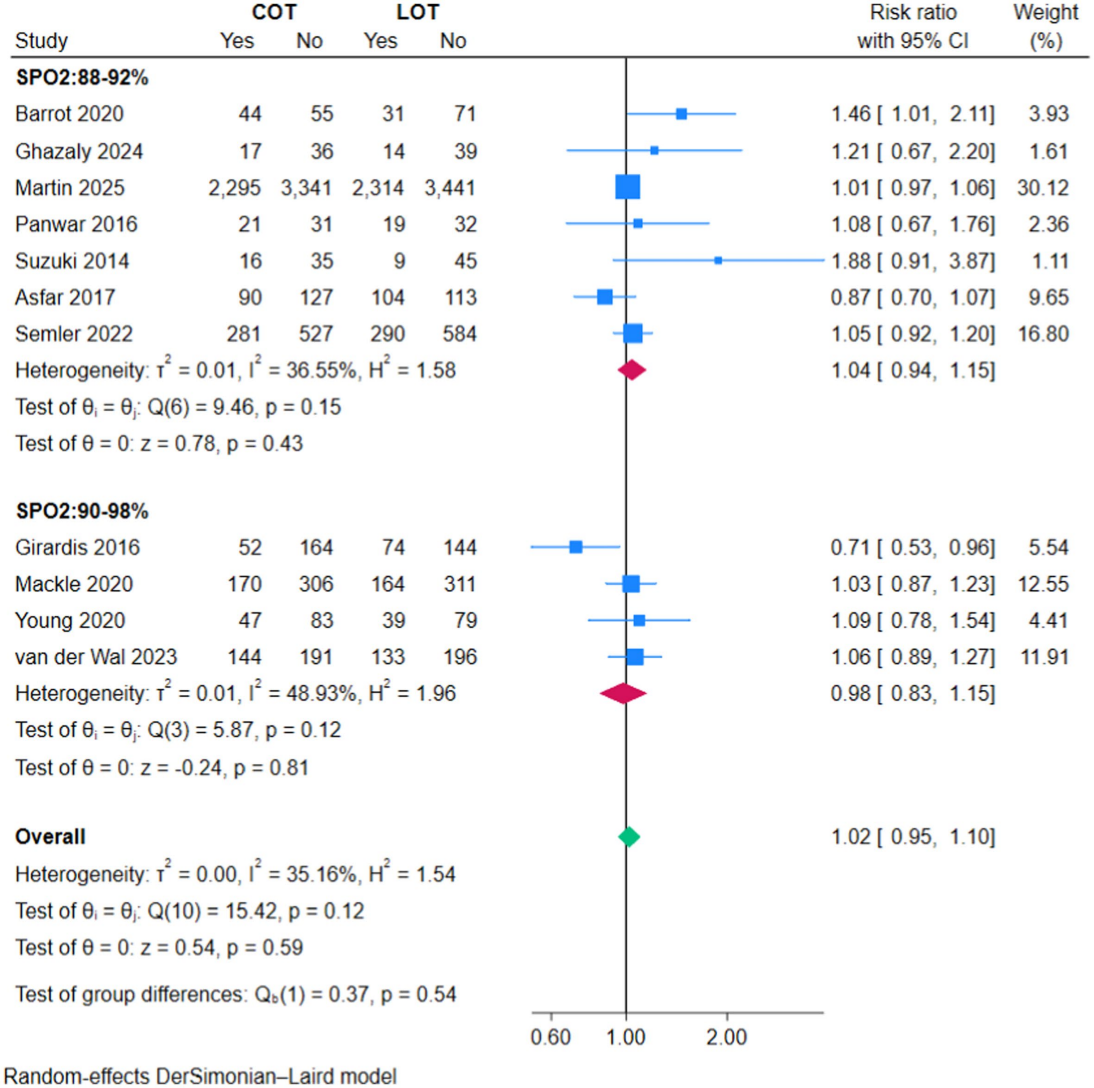
Forest plot of overall mortality.

**Figure 3:**
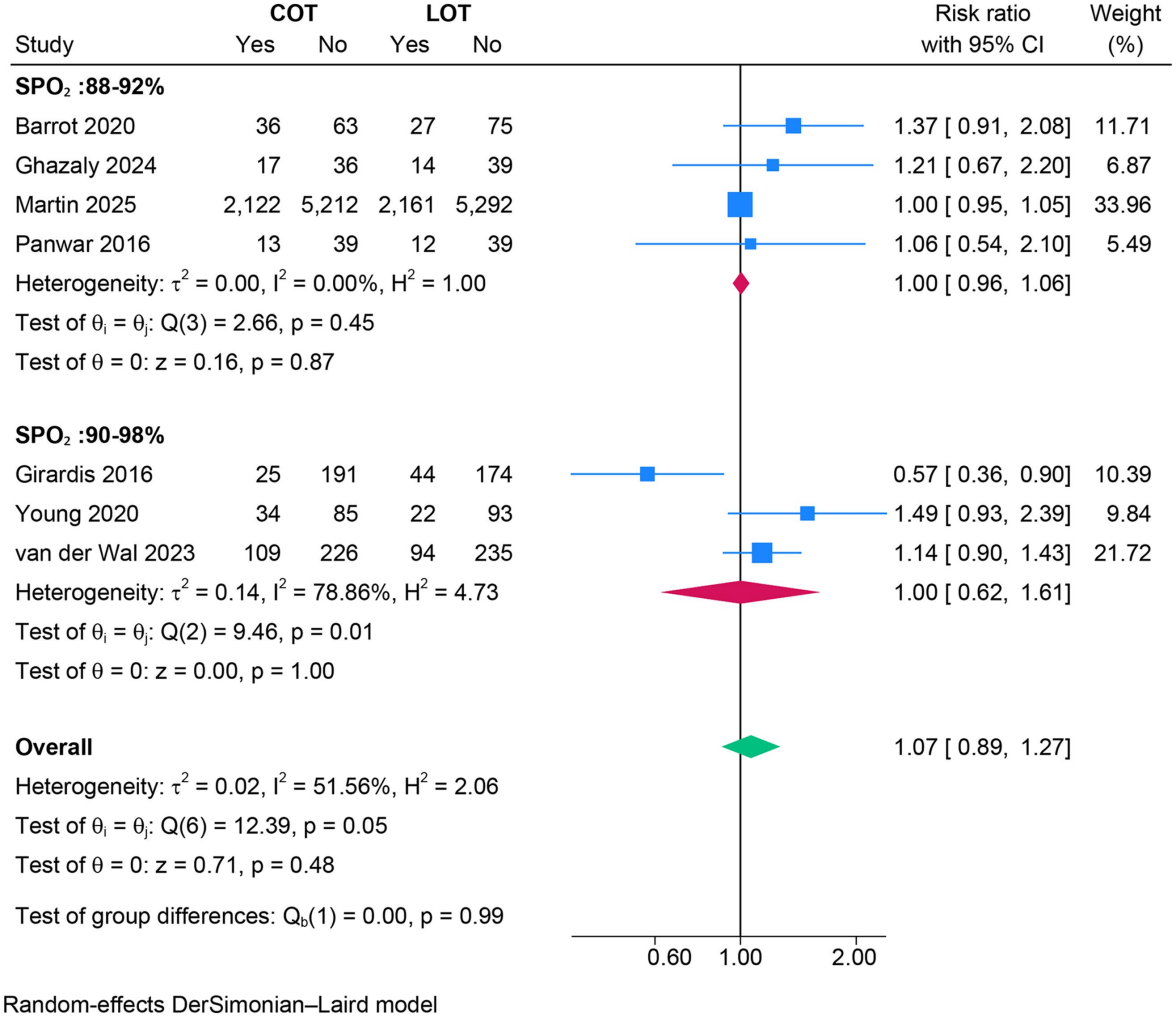
Forest plot of ICU mortality.

**Figure 4:**
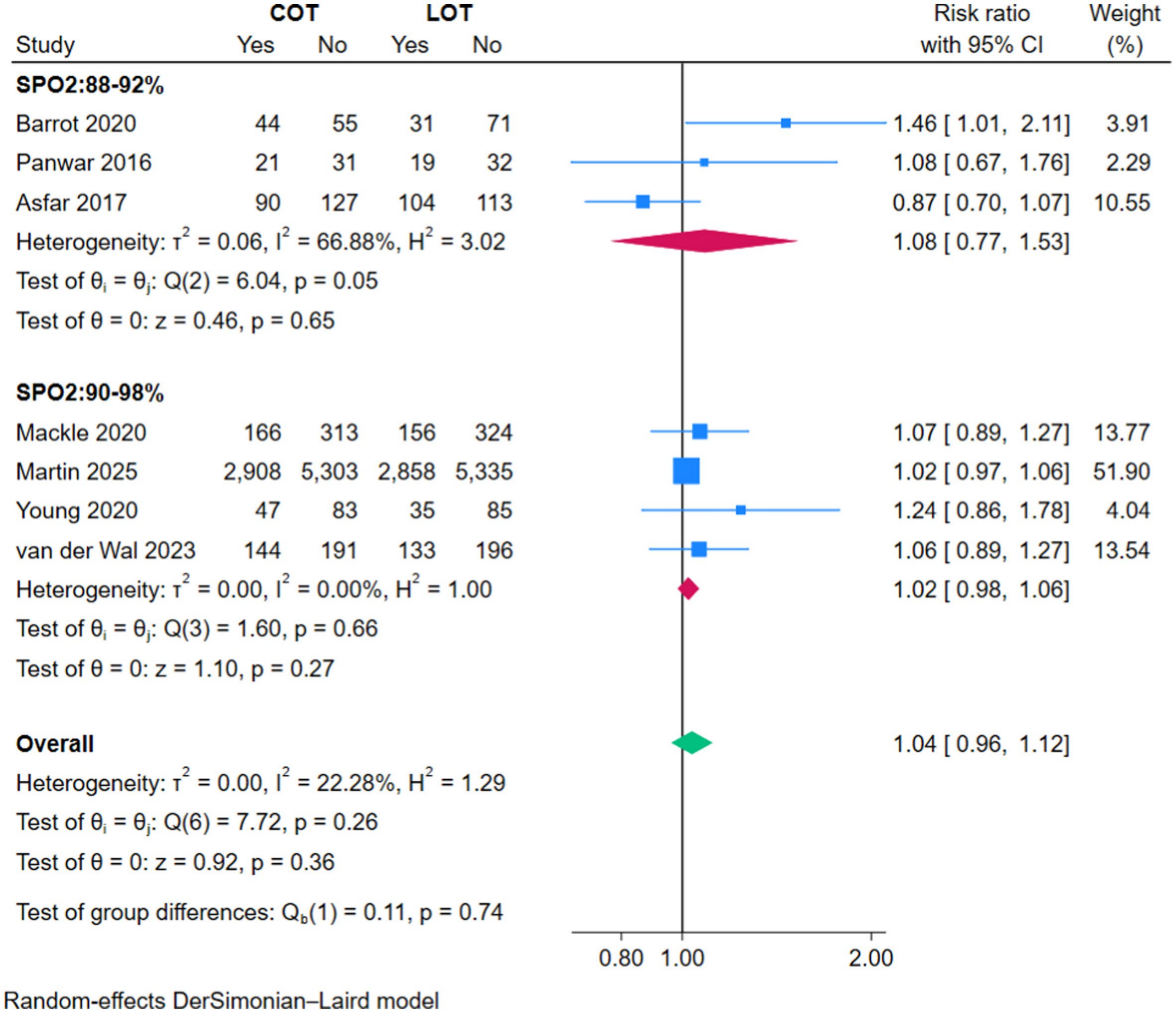
Forest plot of 90-day mortality.

There was no significant difference in overall mortality between COT and LOT (RR 1.02, 95% CI 0.95–1.10; *Z* = 0.54, *p* = 0.59; Figure 2) nor in ICU mortality (RR 1.07, 95% CI 0.89–1.33; *Z* = 0.71, *p* = 0.48; Figure 3) and 90-day mortality (RR 1.04, 95% CI 0.96–1.12; *Z* = 0.92, *p* = 0.36; Figure 4).

### ICU LOS, hospital LOS, and MV hours

**Figure 5:**
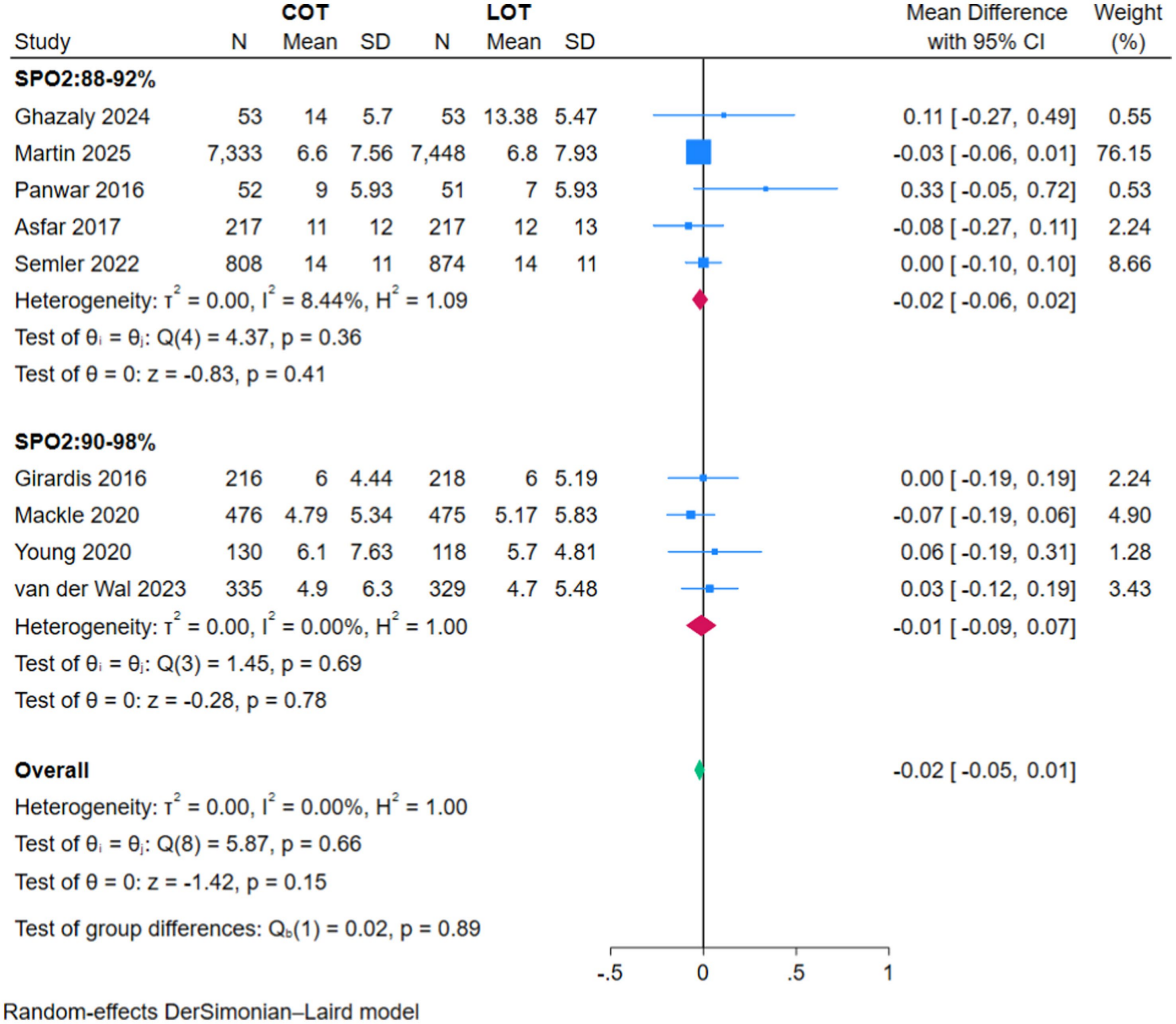
Forest plot of the ICU length of stay.

**Figure 6:**
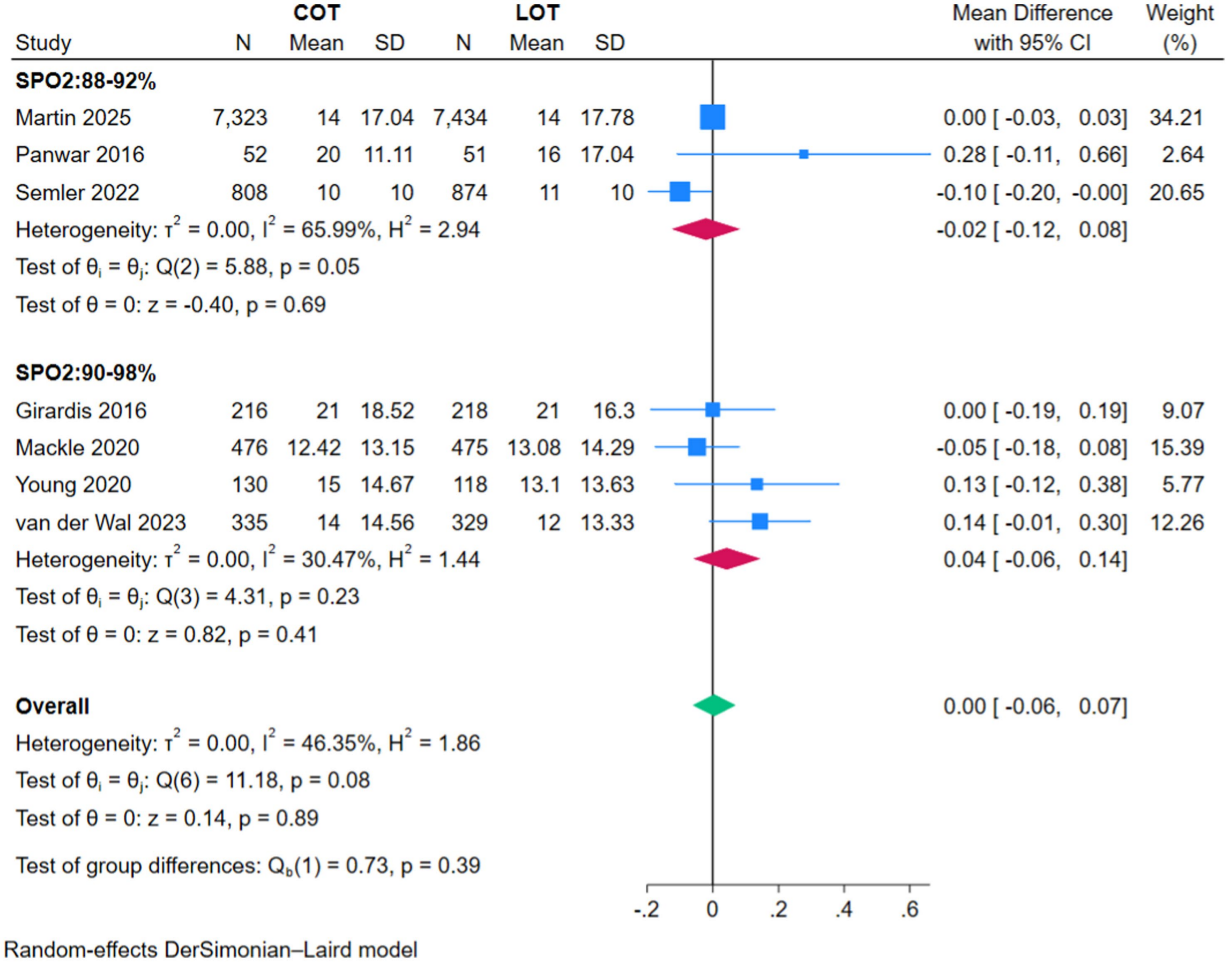
Forest plot of the hospital length of stay.

**Figure 7:**
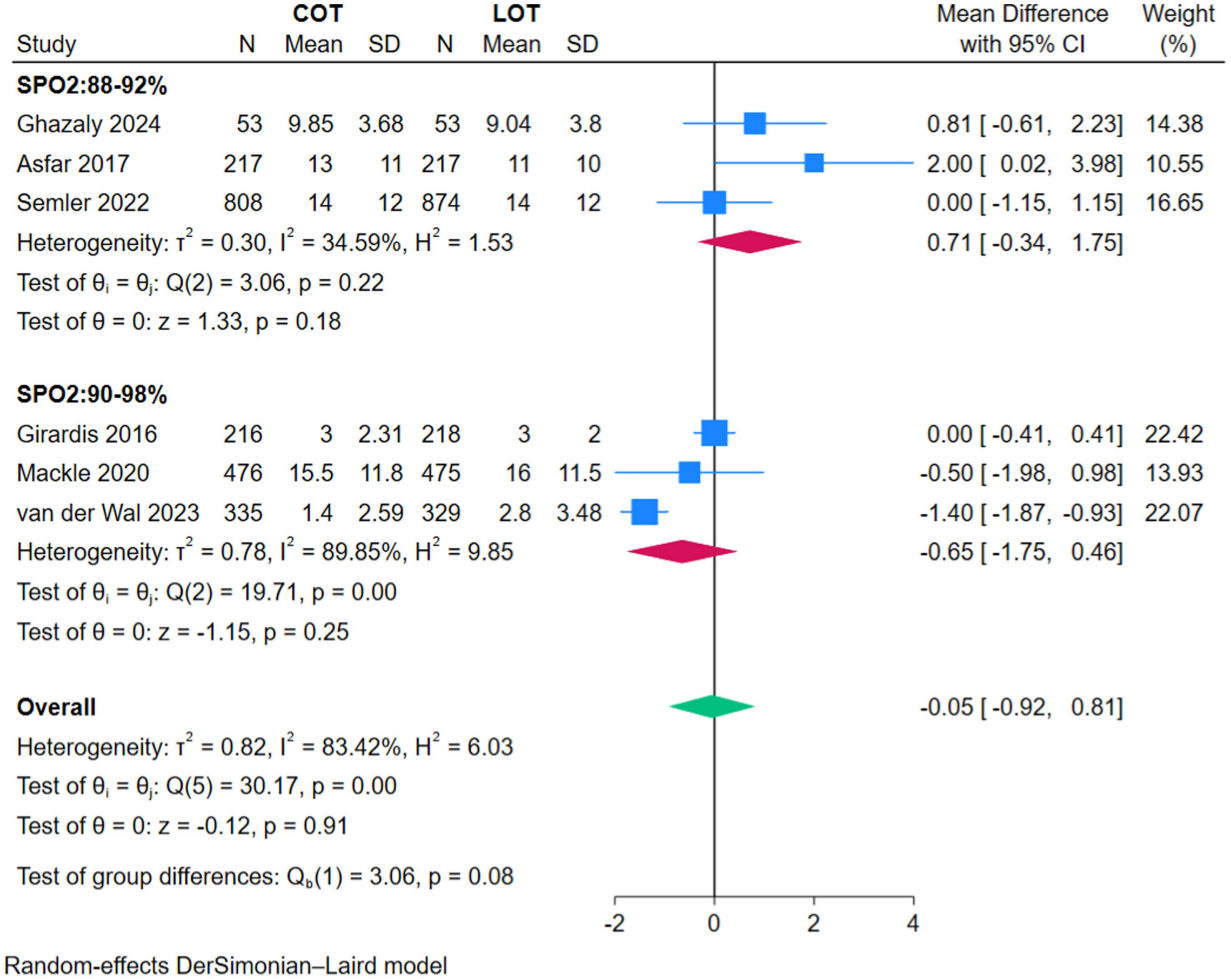
Forest plot of the mechanical ventilation hours.

There was no significant difference in ICU LOS between COT and LOT (MD −0.02, 95% CI −0.05–0.01, *Z* = −1.42, *p* = 0.15; Figure 5), or in hospital LOS (MD 0.00, 95% CI −0.06–0.07, *Z* = 0.14, *p* = 0.89; Figure 6), and MV hours (MD −0.05, 95% CI −0.92–0.81, *Z* = −0.12, *p* = 0.91; Figure 7).

### Subgroup analyses

The subgroup analyses based on the strictness of COT targets (88–92% versus 90–98%) showed no significant effect on primary or secondary outcomes (*p* > 0.05) (Figures 2–7).

### Quality assessment

Overall, the risk of bias was low to moderate, with the exception of blinding and implementation bias. Due to the trial design, it was essentially impossible for clinicians to implement a blinded approach to group-based therapy. Trials were considered to have a low risk of blinding bias if either the clinician or one of the outcome assessors was blinded. A study was assessed as having a high risk of bias due to the potential for COT to increase the risk of serious adverse events, thereby potentially skewing estimates of treatment efficacy (17) (Supplementary Figures S1, S2).

### Publication bias

The Egger’s test was used to assess publication bias among the included studies. No significant publication bias was seen in overall mortality, ICU mortality, 90-day mortality, ICU LOS, hospital LOS, or MV hours (Egger’s test, *p* > 0.05).

## Discussion

This updated systematic review and meta-analysis, which incorporates the landmark UK-ROX trial (13), provides the most precise estimate to date regarding the effects of COT versus LOT in a broad population of MV patients. The primary finding—that COT does not significantly reduce overall mortality, ICU mortality, 90-day mortality, ICU LOS, hospital LOS, or MV hours compared with LOT—remained robust across all analyses. This robustness underscores that the conclusion is not merely an artifact of a single large trial but a consistent signal across the entirety of the current evidence base.

The present meta-analysis was therefore conducted to address two critical gaps. First, by incorporating the UK-ROX trial and other recent RCTs. Second, by conducting predefined subgroup analyses stratified by the strictness of the COT target (88–92% versus 90–98%), which has not been systematically examined previously. This allows this study to assess whether the choice of SpO₂ threshold modifies the treatment effect. Furthermore, these findings suggest that, based on the existing evidence, routine use of COT does not confer a mortality benefit in MV adult patients. However, given the clinical and methodological heterogeneity among the included studies and the potential for future research to refine these estimates, this conclusion should be interpreted with caution. Moreover, the consistent null effect across both the target ranges suggests that clinicians need not adhere to a specific lower threshold, supporting a shift toward individualized oxygen therapy.

The principal implication of this study is that a universal, protocolized COT strategy cannot be recommended for all MV patients. Instead, clinicians should focus on avoiding both severe hypoxemia and hyperemia. This review cannot dictate a single optimal SpO₂ target, but it liberates clinicians from feeling compelled to aggressively titrate FiO₂ to a stringent lower target for fear of missing a mortality benefit. The key to future progress lies in individualization. An RCT involving 726 COVID-19 patients with severe hypoxemia showed that using lower oxygen targets conferred therapeutic advantages (26). Machine learning researchers have suggested that MV patients with acute brain injury have lower mortality with lower SpO_2_ targets, whereas those with sepsis have lower mortality with higher SpO_2_ targets (27). A meta-analysis of patients post-cardiac arrest showed that COT was associated with a significant reduction in mortality (28). One of the key pathophysiological sequelae post-cardiac arrest is hypoxic–ischemic encephalopathy (HIE) following ischemia–reperfusion injury. At this point, excessive oxygen in the circulation after the recovery of cardiac output may be harmful to the brain (29). Therefore, the most important precaution for clinicians is to consider the patient’s underlying pathophysiology. Individualizing therapy based on this review involves using its findings as a foundation—establishing that no single strategy is universally superior—while remaining vigilant for patient-specific factors and future evidence that can guide more personalized targets.

It is also important to acknowledge that these findings do not definitively rule out a potential benefit of conservative oxygenation in specific, yet-unidentified subgroups or with different oxygenation thresholds than those analyzed. The consistent null effect in this broad population should not discourage further research into more personalized approaches, as larger or more homogeneous populations may yet reveal a signal.

This study has several limitations. First, clinical heterogeneity exists in patient populations, co-interventions, and the precise implementation of oxygen targets. In some studies, the SpO₂ threshold for the COT group ranged from 90 to 98% (19, 20, 23, 24), whereas in others it was 88–92% (13, 17, 18, 21), potentially diluting the physiological contrast between the intervention groups. The subgroup analyses suggest that these factors, within the ranges studied, did not significantly alter the overall conclusion. Second, most trials used SpO₂ rather than PaO₂ for titration. While SpO₂ is clinically pragmatic, it may not fully reflect true arterial oxygenation, especially in critically ill patients with poor peripheral perfusion, potentially introducing misclassification bias. Finally, the included trials were heterogeneous in their primary endpoints; some were powered by mortality, while others focused on ventilator-free days or organ failure scores. This limits the strength of mortality conclusions from individual studies.

## Conclusion

This meta-analysis found no significant benefits or harms from COT compared with LOT. COT in MV patients did not reduce overall mortality, ICU mortality, 90-day mortality, or MV hours. The absence of overall benefits from COT in this broad population does not preclude the possibility that selected subgroups or different target ranges could prove advantageous.

## Data Availability

The datasets used and/or analysed during the current study are available from the corresponding author on reasonable request.

## Supplementary material

SUPPLEMENTARY FIGURE S1 Risk of bias graph of included studies.

SUPPLEMENTARY FIGURE S2 Risk of bias summary of included studies.
