# Supplementary figures and images for: Conservative versus liberal oxygen therapy for mechanically ventilated patients: a systematic review and meta-analysis of randomized controlled trials
Source: Front Med (Lausanne). 2026 Apr 24;13:1697749. doi: 10.3389/fmed.2026.1697749 (PMC13153105; doi:10.3389/fmed.2026.1697749)

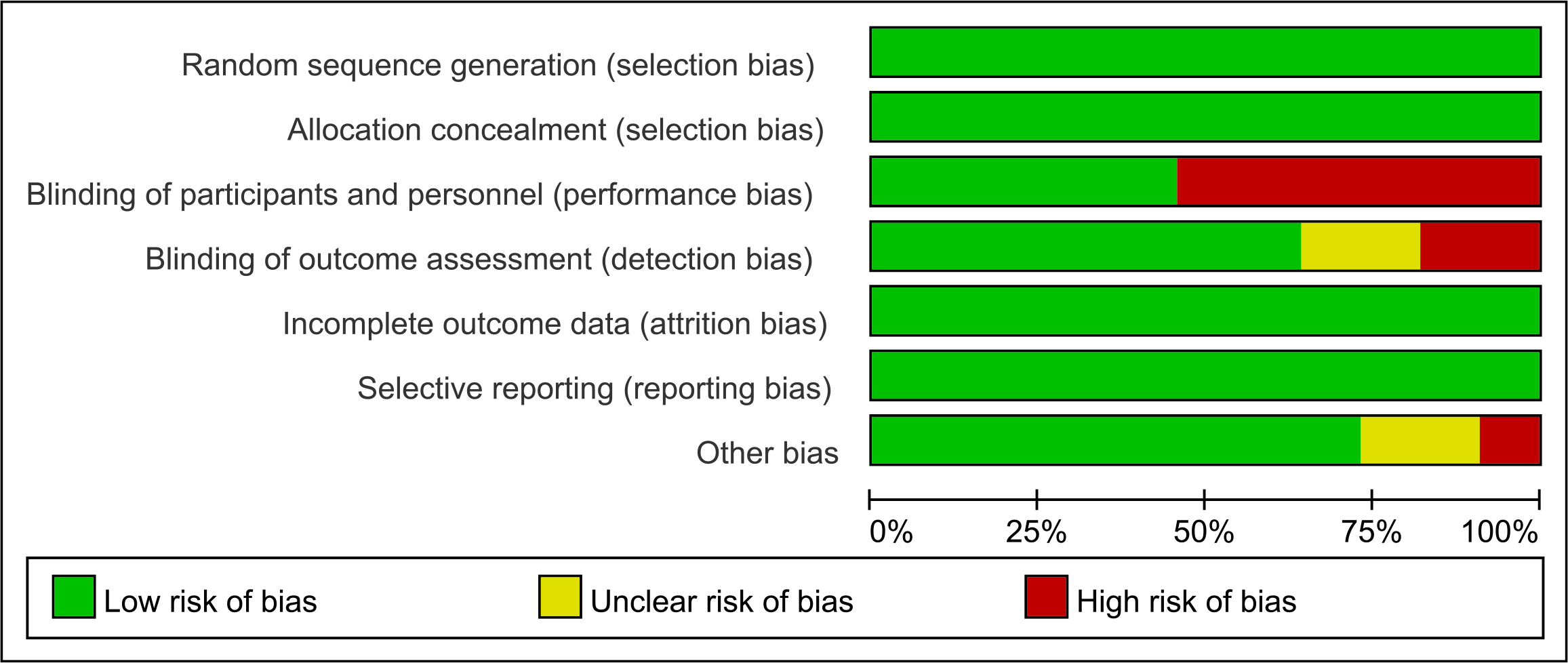

Supplement: SUPPLEMENTARY FIGURE S1 — Risk of bias graph of included studies. [file Image_1.TIF]

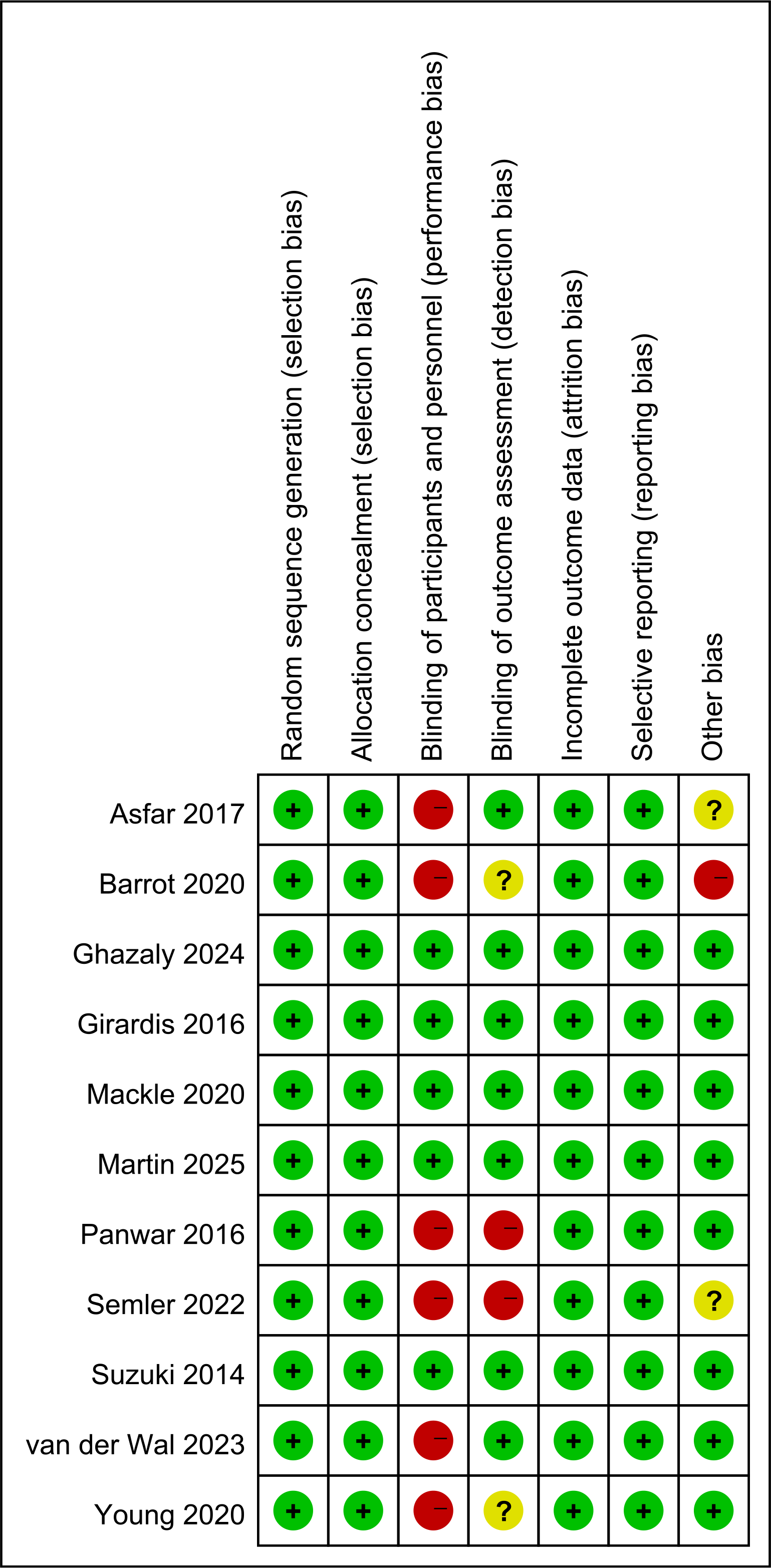

Supplement: SUPPLEMENTARY FIGURE S2 — Risk of bias summary of included studies. [file Image_2.TIF]
