# Supplementary material for: Conservative versus liberal oxygen therapy for mechanically ventilated patients: a systematic review and meta-analysis of randomized controlled trials
Source: Front Med (Lausanne). 2026 Apr 24;13:1697749. doi: 10.3389/fmed.2026.1697749 (PMC13153105; doi:10.3389/fmed.2026.1697749)
Supplement: Supplementary file 3 [file Table_1.DOCX]

Table S1 Literature search strategy

**1.Pubmed**

| Search number | Query | Results |
| --- | --- | --- |
| #1 | "conservative oxygen therapy"[Title/Abstract] OR "COT"[Title/Abstract] OR "lower oxygenation targets"[Title/Abstract] OR "lower oxygenation target"[Title/Abstract] OR "liberal oxygen therapy"[Title/Abstract] OR "LOT"[Title/Abstract] OR "higher oxygenation targets"[Title/Abstract] OR "higher oxygenation target"[Title/Abstract] OR "usual oxygen therapy"[Title/Abstract] | 41,838 |
| #2 | "respiration, artificial"[MeSH Terms] | 94,426 |
| #3 | "mechanical ventilation"[Title/Abstract] OR "mechanical ventilations"[Title/Abstract] OR "artificial respiration"[Title/Abstract] OR "ventilator"[Title/Abstract] | 93,700 |
| #4 | #2 OR #3 | 151,347 |
| #5 | "Randomized Controlled Trial"[Publication Type] | 646,273 |
| #6 | "randomized controlled trial"[Title/Abstract] OR "RCT"[Title/Abstract] OR "clinical trials"[Title/Abstract] OR "controlled clinical trials"[Title/Abstract] OR "randomized controlled clinical trial"[Title/Abstract] OR "Trials"[Title/Abstract] | 966,771 |
| #7 | #6 OR #7 | 1,472,261 |
| #8 | #1 AND#4 AND #7 | 84 |

**2.Embase**

| Search number | Query | Results |
| --- | --- | --- |
| #1 | ''conservative oxygen therapy':ab,ti OR 'cot':ab,ti OR 'lower oxygenation targets':ab,ti OR 'lower oxygenation target':ab,ti OR 'liberal oxygen therapy':ab,ti OR 'lot':ab,ti OR 'higher oxygenation targets':ab,ti OR 'higher oxygenation target':ab,ti OR 'usual oxygen therapy':ab,ti | 68,226 |
| #2 | 'artificial ventilation'/exp | 34,694 |
| #3 | 'mechanical ventilation':ab,ti OR 'mechanical ventilations':ab,ti OR 'artificial respiration':ab,ti OR 'ventilator':ab,ti | 155,917 |
| #4 | #2 OR #3 | 380,015 |
| #5 | 'randomized controlled trial'/exp | 1,101,970 |
| #6 | 'randomized controlled trial':ab,ti OR 'rct':ab,ti OR 'clinical trials':ab,ti OR 'controlled clinical trials':ab,ti OR 'randomized controlled clinical trial':ab,ti OR 'trials':ab,ti | 1,386,513 |
| #7 | #6 OR #7 | 2,166,703 |
| #8 | #1 AND#4 AND #7 | 229 |

**3.Cochrane**

| Search number | Query | Results |
| --- | --- | --- |
| #1 | (Conservative Oxygen Therapy):ti,ab,kw OR (COT):ti,ab,kw OR (COT):ti,ab,kw OR (Lower Oxygenation Targets):ti,ab,kw OR (Lower Oxygenation Target):ti,ab,kw OR (liberal oxygen therapy):ti,ab,kw OR (LOT):ti,ab,kw OR (Higher Oxygenation Targets):ti,ab,kw OR (higher oxygenation target):ti,ab,kw | 3,888 |
| #2 | (Mechanical Ventilation):ti,ab,kw OR (Mechanical Ventilations):ti,ab,kw OR (Artificial Respiration):ti,ab,kw OR (ventilator):ti,ab,kw | 23,498 |
| #3 | (Randomized Controlled Trial):ti,ab,kw OR (RCT):ti,ab,kw OR (Clinical Trials):ti,ab,kw OR (Controlled Clinical Trials):ti,ab,kw OR ( randomized controlled clinical trial):ti,ab,kw OR (Trials):ti,ab,kw | 935,842 |
| #4 | #1 AND #2 AND #3 | 151 |

**4.Web of science**

| Search number | Query | Results |
| --- | --- | --- |
| #1 | TS=(Conservative Oxygen Therapy or COT or Lower Oxygenation Targets or Lower Oxygenation Target or liberal oxygen therapy or LOT or Higher Oxygenation Targets or higher oxygenation target or usual oxygen therapy ) | 369,246 |
| #2 | TS=(artificial ventilation or Mechanical Ventilation or Mechanical Ventilations or Artificial Respiration or ventilator ) | 228,350 |
| #3 | TS=(Randomized Controlled Trial or RCT or Clinical Trials or Controlled Clinical Trials or randomized controlled clinical trial or Trials ) | 3,215,406 |
| #4 | #1 AND #2 AND #3 | 498 |
